# The Marine Biomonitor *Mytilus galloprovincialis*: A Study on Microplastics as a Vector for Heavy Metals Pollution in Sardinia (Italy)

**DOI:** 10.3390/foods15193417

**Published:** 2026-09-24

**Authors:** Gabriella Piras, Alessandro Cau, Domenico Meloni, Bruna Vodret, Monica Cangini, Rita Melillo, Sara Salza, Monica Molotzu, Simona Cau, Giuseppa Lorenzoni, Tiziana Tedde, Riccardo Bazzardi, Maurizio Cossu, Andrea Sanna, Sonia Dall’Ara, Sebastiano Virgilio

**Affiliations:** 1 Struttura Complessa di Controllo Microbiologico e Ispezione degli Alimenti, Istituto Zooprofilattico Sperimentale della Sardegna, Via Duca degli Abruzzi 8, 07100 Sassari, Italy; gabriella.piras@izs-sardegna.it (G.P.); rita.melillo@izs-sardegna.it (R.M.); sara.salza@izs-sardegna.it (S.S.); monica.molotzu@izs-sardegna.it (M.M.); pina.lorenzoni@izs-sardegna.it (G.L.); tiziana.tedde@izs-sardegna.it (T.T.); riccardo.bazzardi@izs-sardegna.it (R.B.); sebastiano.virgilio@izs-sardegna.it (S.V.); 2 Dipartimento di Scienze della vita e dell’ambiente, Università degli Studi di Cagliari, Via Tommaso Fiorelli 1, 09126 Cagliari, Italy; alessandro.cau@unica.it; 3 Dipartimento di Medicina Veterinaria, Università degli Studi di Sassari, Via Vienna 2, 07100 Sassari, Italy; 4 Struttura Complessa di Chimica, Istituto Zooprofilattico Sperimentale della Sardegna, Via Duca degli Abruzzi 8, 07100 Sassari, Italy; bruna.vodret@izs-sardegna.it (B.V.); simona.cau@izs-sardegna.it (S.C.); maurizio.cossu@izs-sardegna.it (M.C.); andrea.sanna@izs-sardegna.it (A.S.); 5 Fondazione Centro Ricerche Marine, Laboratorio Nazionale di Riferimento per il Monitoraggio delle Biotossine Marine, Viale Amerigo Vespucci 2, 47042 Cesenatico, Italy; monica.cangini@centroricerchemarine.it (M.C.); sonia.dallara@centroricerchemarine.it (S.D.)

**Keywords:** *Mytilus galloprovincialis*, aquaculture, microplastics, heavy metals

## Abstract

The main objective of the present study was to assess the contamination level and nature of microplastics and their role as vectors of trace elements in Mediterranean mussels (*Mytilus galloprovincialis*). A total of 39 samples were collected from four farming areas (1–4) located in Sardinia (Italy) and selected according to the principles of the risk assessment. The concentrations of 22 trace elements (Be, Tl, Sb, Ag, Hg, Th, U, Sn, Co, Cd, Li, Mo, Cr, Ba, V, Ni, Pb, Se, Mn, Cu, As, Zn) in mussels and plastic particles extracted from mussels was determined by inductively coupled plasma mass spectrometry (ICP-MS/MS). The isolated plastic particles were characterized by Fourier Transform Infrared Spectroscopy (µ-FTIR). All samples were compliant with the European Union official limits set for Hg, Cd and Pb (Reg. UE 915/2023). Plastic particles were identified in all the samples with a total of 1007 isolated microfibers. Qualitative characterization of microplastics showed that natural polymers predominated, accounting for over 85% of the isolated microfibers. Cellulose was the most abundant polymer (55.29%), followed by cotton (30.59%). Synthetic polymers were present only as a minor fraction (~15%). These results support the use of *Mytilus galloprovincialis* as a reliable bioindicator of microplastics pollution. Heavy metal concentrations associated with microplastics ingested by *Mytilus galloprovincialis* were markedly lower than those measured directly in the edible tissues of the mussels, highlighting that microplastics likely contribute minimally as vectors of metals to human consumers. The results of the present study allowed for a comprehensive evaluation of both microplastics contamination and the distribution of heavy metals in mussels reared in Sardinia, providing a robust framework for subsequent risk assessment studies.

## 1. Introduction

The widespread presence of plastic contaminants in the marine environment is considered a global threat to marine and terrestrial ecosystems [1]. Once in the marine environment, plastic undergoes photodegradation and fragmentation, breaking down into small plastic particles (<5 mm) called microplastics (MPs) that pervade the water columns and sediments [2]. Plastic particles make up 60–90% of ocean debris, mainly consisting of microplastics mainly resulting from the fragmentation of larger objects [3]. MPs come in a wide range of sizes (0.1–5000 µm), shapes (pellets, fibers, fragments, etc.), colors (black, blue, transparent, etc.), and polymer composition such as polyethylene (PE), polypropylene (PP), polystyrene (PS), polyethylene terephthalate (PET) and polyvinyl chloride (PVC) [4]. Their distribution and accumulation have been widely observed in aquatic, terrestrial, and atmospheric environments, and their presence has been reported in various food products for human consumption [5,6,7,8,9]. It is well known that the absorption of MPs causes negative effects in living organisms, and this is an emerging environmental issue. These tiny particles, because of their physico-chemical properties, surface characteristics, large specific surface area, and hydrophobic nature, have the potential to adsorb other harmful contaminants from their surroundings [10,11].

Plastics, in their different formulations, often include toxic chemical additives to achieve specific mechanical properties (for example, phthalates for durability) and aesthetic qualities (for example, pigments for color), or toxic chemicals from the marine environment can be adsorbed on their surface [2]. Several studies have highlighted their role as carriers of heavy metals, which they can contain in varying amounts, representing a potential threat to the environment and biota due to their toxicity [12,13]. MPs and heavy metals, although representing two distinct classes of pollutants, show an important interaction: it is believed that ingesting MPs can release and transfer heavy metals and their compounds throughout the entire food chain, and their bioaccumulation can occur through biomagnification [14].

Heavy metals are widespread in aquatic ecosystems: some peculiar characteristics such as high toxicity, multiple origins, and refractory properties make them an environmental and health problem [15,16]. Their presence can lead to the bioaccumulation of toxic metals in aquatic species, with long-term negative impacts on human health [17,18]. Bivalve mollusks are exposed to MPs through the water column [19,20,21] and, because of their high bioaccumulation capacity [22] and geographic distribution [23], they are used as sentinel organisms, especially in the marine environment, acting as good bioindicators not only for plastic particle contamination [24] but also for monitoring heavy metals [25]. Bivalve mollusks usually absorb metals from the water through their gills via simple diffusion, and from there they further spread to other tissues through the hemolymph. They are then transported to the digestive gland where they accumulate before being delivered to other tissues [22].

The Mediterranean Sea is heavily affected by plastic pollution and is considered one of the major accumulation areas for MPs [26,27,28,29], mainly due to its semi-enclosed nature and the high human population density along its coasts [30]. Even though it covers less than 1% of the Earth’s ocean surface, it is estimated to hold over 7% of the global MPs. Urbanization, tourism, shipping, fishing, and poor waste management all contribute to the introduction of large amounts of plastic waste into the Mediterranean [2].

The bivalve mollusk *Mytilus galloprovincialis*, commonly known as the Mediterranean mussel, holds a prominent place in the marine ecosystems of the Mediterranean [24]. It has a wide distribution range and, being an edible species, has significant commercial value [2,31]. *Mytilus galloprovincialis* stands out as an important marine biomonitor species, reflecting the impact of MPs pollution on marine organisms and potentially on human health through consumption [32].

The objectives of the present study were (a) to evaluate the prevalence of MPs and heavy metals in *Mytilus galloprovincialis* reared in Sardinia; (b) to characterize the isolated MPs through the use of Fourier Transform Infrared Microscopy (μ-FTIR); (c) to assess the role of MPs as vectors of heavy metal pollution; and (d) to contribute to the assessment of consumer exposure associated with the consumption of seafood.

## 2. Materials and Methods

### 2.1. Study Area, Collection, Transport, and Storage of Samples

From May 2023 to April 2024, a total of 39 samples of Mediterranean mussels (*Mytilus galloprovincialis*), weighing about 1 kg each, were collected during three sampling sessions (S1–S3) using a random sampling procedure from four farming areas located in different parts of Sardinia (Italy). A total of 13 samples were taken during each of the three samplings: S1 (May–August 2023); S2 (September–December 2023) and S3 (January–April 2024). In order to ensure statistical, spatial, and temporal significance across the different aquaculture areas, several risk factors were taken into consideration to assess the possible exposure of *Mytilus galloprovincialis* to MPs and heavy metals: (a) presence of industrial settlements with large-scale use of plastic products; (b) presence of large urban centers with high touristic presence; (c) presence of seaports; (d) presence of farming and livestock breeding; and (e) territorial representativeness. The four farming areas (1–4) were selected based on the presence of these risk factors (Figure 1). In each farming area, the following sampling stations were identified: (1) Olbia farming area: Cala Saccaia, Isola del Cavallo-Mezzocammino, Seno Cocciani, Golfo di Cugnana; (2) Tortolì farming area: Stagno di Tortolì; (3) Cagliari farming area: Laguna di Santa Gilla, Stagno di San Giovanni, Stagno di Feraxi; (4) Oristano farming area: Capo San Marco, Stagno di Corru Mannu, Torregrande, Terza Peschiera. The samples were tagged with the code of the farming area and of the sampling point and were sent straight under refrigeration to the laboratories of the Istituto Zooprofilattico Sperimentale della Sardegna in Sassari (Italy). The samples were handled carefully to minimize physical damage during transportation. Each sample of *Mytilus galloprovincialis* was divided into two sub-samples: the first sub-sample was analyzed as-is to perform chemical analysis. The second sub-sample was frozen at −18 °C to evaluate the possible presence of microplastics and their role as a vector for heavy metal pollution.

### 2.2. Assessment of Biometric Characteristics of Mytilus galloprovincialis

For each specimen of *Mitylus galloprovincialis*, the total length and width of the mussel in centimeters (cm) and the weights of the whole specimen and of the soft tissue, expressed in grams, were recorded in a dedicated form.

### 2.3. Quantification of Heavy Metals in Mytilus galloprovincialis

A total of twenty-two heavy metals (Be, Tl, Sb, Ag, Hg, Th, U, Sn, Co, Cd, Li, Mo, Cr, Ba, V, Ni, Pb, Se, Mn, Cu, As, Zn) were quantified in Mediterranean mussels. The choice of heavy metals to include in the study involved two steps. Firstly, an initial assessment concerning the food safety of bivalve mollusks was carried out, aimed at determining the heavy metals regulated in the EU: Pb, Cd and Hg [33]. In addition, other heavy metals not included in the EU regulation but of toxicological relevance were also analyzed, as well as other heavy metals useful for characterizing the environmental conditions of the study areas. The first sub-sample was checked to make sure it was intact and suitable for analysis. After that, the specimens were carefully washed with deionized water to remove surface residues, sediments, and any debris sticking to the shells, to avoid contamination in the next steps. Then, the shells were opened and the edible tissue was manually separated using inert tools to reduce the risk of metal contamination. The collected tissue mass was brought to a quantity of about 50 g, representative of the entire sub-sample of mussels. The edible tissue was then homogenized using a BÜCHI Mixer B-400 homogenizer (BÜCHI Labortechnik, Flawil, Switzerland) to ensure maximum uniformity of the matrix and guarantee the representativeness of the analytical sample. An aliquot of about 1 g was taken for the mineralization phase, using calibrated and properly decontaminated instruments. Mineralization of the homogenized sample was carried out under controlled temperature and pressure conditions using a CEM Discover^®^ SP-D Microwave Synthesizer (CEM Corporation, Matthews, NC, USA). The solutions of the mineralized samples were first diluted to a volume of 50 mL with water and then, before instrumental analysis, further diluted at a ratio of 1 to 5 mL.

Trace elements were determined by inductively coupled plasma-mass spectrometry (ICP-MS/MS) on an Agilent 8800 Triple Quad ICP-MS (Agilent, Santa Clara, CA, USA) equipped with a collision cell and two mass selection quadrupoles in accordance with US EPA 6020B for instrumental analysis and US EPA 3052 for sample preparation. The concentrations of heavy metals in the reading solutions were determined by interpolating the reading signal with multi-level calibration curves. In each analytical session, the accuracy of the analytical procedure was verified by analyzing Certified Reference Materials (Mussel Tissue-SRM) 2976 from National Institute of Standard and Technology. For Quality Control (QC), reagent blanks were also determined in each series of the analysis. The analytical methods were validated according to the UNI EN ISO 17025/2017 standard [34]. The limits of detection (LOD) and quantification (LOQ) were determined based on the standard deviation (σ) calculated from 20 independent blank measurements. The LOD and LOQ were calculated as 3σ and 10σ, respectively. For all the elements analyzed, the limit of quantification (LOQ) was <0.010 mg·kg^−1^, while the limit of detection (LOD) was <0.003 mg·kg^−1^.

### 2.4. Sample Preparation for the Identification and Quantification of Plastic Particles

In order to ensure representative significance, three specimens of *Mytilus galloprovincialis* were randomly selected from the second sub-sample. Each mussel was analyzed separately, rinsed with prefiltered distilled water, and placed in 150 mm diameter, 20 mm high glass Petri dishes, covered with a sheet of aluminum foil. The edible part of the mollusk (flesh) was dissected using scissors, rinsed with prefiltered distilled water, and placed into a 200 mL glass bottle, previously weighed empty, covered with aluminum foil to minimize contamination from MPs in the air. The bottle was then weighed to determine the sample weight by subtraction. Each sample was digested using a highly alkaline reagent, potassium hydroxide (KOH, Carlo Erba, Milan, Italy) at 10% in a 1:5 ratio (*w*/*v*) and incubated at 40 °C for 48 h. The contamination of water and solutions used for the KOH 10% (*w*/*v*) digestion was assessed through the preparation of a procedural blank consisting of 30 mL of 10% KOH and a field blank consisting of a cellulose nitrate filter placed on a 60 mm diameter, 20 mm high glass Petri dish under a laminar flow hood during the processing stages in order to passively assess the presence of MPs in the environment. The following density separation step was carried out using a saturated NaCl solution (Sodium chloride, Sigma-Aldrich, St Louis, MO, USA) to remove as much of the organic component as possible and to allow the recovery of plastic particles that tend to float on the surface of the 100 mL volume solution. This step was repeated three consecutive times and was followed by a 5 min decantation phase, after which, the samples were filtered. The filtration was carried out on the supernatant, where any plastic particle present were dispersed, through cellulose nitrate filters with a porosity of 8 µm and 47 mm in diameter (Sartorius, Varedo, Italy) by a vacuum pump (Thermo Savant VLP200 Valu Pump, Thermo Fisher Scientific™, Waltham, MA, USA). The labeled filters were placed on 60 mm diameter, 20 mm high glass Petri dishes to prevent contamination and left to dry at room temperature for 12–24 h. The Petri dishes were sent to the laboratories of the Dipartimento di Scienze della vita e dell’ambiente in Cagliari (Italy) for identification and quantification of plastic particles.

### 2.5. Identification and Quantification of Plastic Particles

Each sample was visually examined by a stereo microscope Zeiss Stemi DV4 (Carl Zeiss, Oberkochen, Baden-Württemberg, Germany) with magnifications from 5× to 20× to identify plastic particles based on their morphological features (shape, color, size). For each visual sorting session, controls were set up to monitor contamination, as described below in Section 2.7. The plastic particles present in procedural and field blanks were analyzed following the same protocol described for *Mytilus galloprovincialis* samples. Plastic particles lengths were determined by extracting the photos obtained under the stereo microscope at 5× magnification into AutoCAD software (version 2026), using the “scale”, “spline” and “pedit” functions to bring the images to a 1:1 scale and create a measurable shape for each individual MP.

### 2.6. Characterization of MPs by Fourier Transform Infrared Microscopy (μ-FTIR)

For each *Mytilus galloprovincialis* specimen, the number of plastic particles and the total sum of their lengths were recorded. A representative sub-sample (10%) of all the collected plastic particles, consisting of the larger ones (*n* = 100) and therefore easier to handle, was taken, isolated, and fixed on a suitable substrate for polymer analysis using Fourier Transform Infrared Spectroscopy (μ-FTIR) with the Nicolet™ iN10 MX instrument (Thermo Fisher Scientific™) in ATR (attenuated total reflectance) mode, using a germanium tip with a spatial resolution down to 10–20 µm. The spectra were acquired over the spectral range of 800 to 4000 cm^−1^, with 64 scans per sample. The spectral resolution was down to 4 cm^−1^. Collected spectra were then analyzed using the OMNIC™ Picta™ software v1.9 and compared to reference spectra of Thermo Fisher Scientific™ libraries (e.g., Microplastics reflection; Hummel Polymer Sample and HR Sprouse Polymers by Transmission) or created in-house. In order to exclude laboratory contamination and confirm the different MPs’ similarities between the major peaks and standard spectra, a match score of 70% or higher was included. The same procedure was applied to items from procedural and field blanks to subtract MPs that could be attributed to environmental contamination from the final count.

### 2.7. Quality Assurance and Quality Control (QA/QC)

To ensure the highest accuracy and reliability of the results, during the handling and processing of the samples, strict measures were carried out to minimize procedural and environmental cross-contamination with airborne MPs or those eventually present in the solvents used [35]. To prevent such contamination, operators wore only bright acid green lab cotton coats and nitrile gloves. All the tools used (knives, tweezers, scissors, flasks, plates, pipettes, etc.) were free of plastic components and were always rigorously washed with demineralized water (PURELAB, option R-7/15, ELGA LabWater, High Wicombe, UK). All operations were carried out under a laminar flow hood equipped with fluorescent orange sleeves [35]. In each sampling session, the contamination level was checked by placing a clean cellulose nitrate filter throughout the duration of the experiment to evaluate the presence of airborne synthetic fibers. The water and the solutions used for digestion (10% KOH (*w*/*v*)) were filtered before use. The total number of plastic particles observed in the procedural and field blanks was subtracted from the total number of plastic particles in the samples [36].

### 2.8. Quantification of Heavy Metals in MPs

A total of twenty-two heavy metals (Be, Tl, Sb, Ag, Hg, Th, U, Sn, Co, Cd, Li, Mo, Cr, Ba, V, Ni, Pb, Se, Mn, Cu, As, Zn) were quantified in samples of MPs. The MPs arrived in the laboratory collected in a slide. The bottom part was made of glass, while the two upper sections were made of plastic rectangles. The MPs were trapped in the interface space between the two materials. The two sections contained MPs extracted from two different specimens taken from the same sampling point. In order to minimize the loss of particulate matter, the slide was placed vertically over the collection tube to allow MPs that detached during the opening of the first sector to fall inside the container. Subsequently, the slide was returned to position and all the samples were washed with five 200 µL microflows of concentrated nitric acid, for a total volume of 1 mL, and rinsed with five microflows of 200 µL of Milli-Q water, for a total volume of 1 mL. At the end of the washing operations, the slides and plastic supports were closed and stored in order to allow subsequent control under the microscope, aimed at verifying the possible presence of residual particles. Mineralization of the MPs samples was carried out under controlled temperature and pressure conditions using a CEM Discover^®^ SP-D Microwave Synthesizer (CEM Corporation). The solutions of the mineralized samples were diluted to a volume of 10 mL with water before instrumental analysis. Trace elements were determined as described in Section 2.3.

## 3. Results and Discussion

### 3.1. Assessment of Biometric Characteristics

The biometric parameters of the analyzed samples are detailed in Table 1. Overall, the examined samples showed an average length of 6.94 ± 0.08 cm, an average width of 3.63 ± 0.04 cm, an average total weight of 19.48 ± 0.76 g, and soft tissue weight of 4.97 ± 0.21 g. During S1, the biometric data showed an average length of 6.99 ± 0.15 cm, an average width of 3.59 ± 0.07 cm, an average weight of 19.01 ± 1.49 g, and a soft tissue weight of 5.37 ± 0.36 g. The biometric data obtained from S2 showed an average length of 6.84 ± 0.30 cm, an average width of 8.44 ± 1.24 cm, an average weight of 18.01 ± 1.19 g, and an average soft tissue weight of 3.72 ± 0.23 g. During S3, the average length of the individuals was 6.99 ± 0.14 cm, the average width 3.70 ± 0.09 cm, the average total weight 21.29 ± 1.19 g, and the soft tissue weight 5.73 ± 0.38 g.

### 3.2. Quantification of Trace Elements in Mytilus galloprovincialis

The concentrations of trace elements in *Mytilus galloprovincialis* are summarized in Table 2. For each farming area, the mean values of the twenty-two heavy metals (Be, Tl, Sb, Ag, Hg, Th, U, Sn, Co, Cd, Li, Mo, Cr, Ba, V, Ni, Pb, Se, Mn, Cu, As, Zn) are reported.

#### 3.2.1. Essential Trace Elements

The concentrations of Co and Mo were pretty consistent across the different farming areas, with higher average values of Co in the Cagliari farming area (0.082 mg·kg^−1^) and Mo in the Olbia farming area (0.186 mg·kg^−1^). These results were in accordance with previous studies carried out in the same species collected from different Sardinian production areas [16,17]. Se showed moderate variability across the different sampling areas, with higher values in the Cagliari sampling area. This distribution matched natural differences in sediment composition and specific minerals found in those areas without exceeding levels considered critical and do not indicate human-caused contamination. Se levels were significantly lower than those detected in mussels collected from several Sardinian production areas [16]. Mn and Cu showed high concentrations in all samples, consistent with its natural abundance in sediments and in the skeletons of many marine organisms [15,16,17,18]. Variations between areas mainly reflect geological differences: higher concentrations were found in the Olbia and Cagliari farming areas. According to previous studies carried out in several bivalve mollusk species sampled in Sardinia, Zn showed the highest concentration among all the considered trace elements with values ranging from 18.875 mg·kg^−1^ (Tortolì farming area) to 31.493 mg·kg^−1^ (Oristano farming area) [15,16,17,18].

#### 3.2.2. Non-Essential Trace Elements

The mean concentrations of Be, Tl, Sb, Ag, Hg were found to be below the method’s limit of quantification (LOQ) (0.010 mg·kg^−1^) in all the farming areas. Th and U were detected in 50% and 85% of the samples, respectively. Both metals showed higher concentrations in the Olbia farming area, with average values of 0.020 mg·kg^−1^ for Th and 0.016 mg·kg^−1^ for U. All the farming areas emerged as clearly contaminated by Sn. The Tortolì farming area showed lower average values (0.022 mg·kg^−1^), while the Olbia farming area showed higher average values (0.070 mg·kg^−1^). Overall, Cd levels remained below the European Union official limits [33], confirming no evidence of significant bioaccumulation in bivalve mollusks reared in Sardinia [15,16,17,18]. Li showed marked variations depending on the farming area. The highest average concentration was recorded in Olbia (0.150 mg·kg^−1^). The concentration of Cr ranged from 0.075 mg·kg^−1^ (Tortolì farming area) to 0.145 mg·kg^−1^ (Cagliari farming area). These results were in accordance with previous studies carried out in the same species collected from different Sardinian production areas [16,17]. Ba showed moderate variability across the different sampling areas, with higher values in the Cagliari sampling area (0.981 mg·kg^−1^). This distribution matched natural differences in sediment composition and specific minerals found in those areas without exceeding levels considered critical and do not indicate human-caused contamination. V did not showed variability across the different sampling areas [16]; on the contrary, Ni showed moderate variability across the different sampling areas, with higher values in the Olbia sampling area (0.640 mg·kg^−1^). The concentration of Ni was significantly higher than those detected in the same species collected from different Sardinian production areas [16]. The concentration of Pb could potentially be influenced by human activities (ports, effluents, anti-fouling paints); however, this study confirmed a generally stable situation [15,16,17,18] and values below the European Union official limits [33]. As shown, higher average values were present in the Tortolì farming area. However, these levels were consistent with the regional geochemical background and do not indicate clear anomalies. The observed concentrations were within the typical ranges of Mediterranean coastal sediments rich in minerals of volcanic or metamorphic origin [15,16,17,18].

### 3.3. Identification and Quantification of Plastic Particles

The results (Table 3) showed a comparison of plastic particles contamination levels in the main coastal areas of Sardinia, looking at differences by geography and over time, using a widely tested and efficient species as a bioindicator, *Mytilus galloprovincialis* [24,29]. All samples were found to be contaminated with plastic particles (100% occurrence), and the results showed that 100% of the isolated items were microfibers (MFs). In total, 1007 MFs were recorded, with an average of 8.99 ± 0.66 items per individual. The maximum number of fibers isolated from a single individual was 40 from the sampling point Isola del Cavallo-Mezzocammino (Olbia farming area), with a minimum of 1. The total length of the fibers collected was 1193.8 mm (average: 10.75 ± 0.9 mm per individual).

#### 3.3.1. Seasonal Effect

During S1, the total number of isolated items was 278 fibers, with an average of 7.13 ± 0.74 fibers per individual. The maximum value was 20 fibers per individual (Santa Gilla 2, Cagliari farming area) and the minimum was 1 (Stagno di Corru Mannu, Oristano farming area). The total length of the collected fibers was 361.3 mm, with an average of 9.5 ± 1.50 mm. A total of 501 fibers were recorded in S2, with an average per individual of 13.9 ± 1.4. The maximum number of fibers per individual was 40 (Golfo di Cugnana, Olbia farming area), with a minimum of 3 (Santa Gilla 2, Cagliari farming area). The total length of collected fibers was 598.2 mm, with an average length of 16.6 ± 1.9 mm. During S3, the total number of fibers was 228, with an average of 6.2 ± 0.7 fibers per individual, a maximum of 18 (Seno Cocciani, Olbia farming area) and a minimum of 1 fiber (Santa Gilla 3, Cagliari farming area). The sum of the lengths of the collected fibers was 234.3 mm, with an average of 6.3 ± 0.9 mm. The trends of both descriptors, i.e., number of particles and total fiber length, over the three samplings of S1 (May–August 2023), S2 (September–December 2023), and S3 (January–April 2024), are shown in Figure 2.

#### 3.3.2. Spatial Analysis

Overall, Capo San Marco (Oristano farming area) stands out as the sampling point with the lowest average number of fibers collected. On the other hand, Isola del Cavallo-Mezzocammino (Olbia farming area) showed both the highest overall abundance and peak fiber accumulation during S2. This peak has also been described, although with lower average abundance, at the sites of Feraxi (Cagliari farming area), Corru Mannu, Terza Peschiera (Oristano farming area) and Tortolì farming area. Such a sudden increase could be due to the September–October time frame, reflecting the effects of the high tourist season. One of the most relevant findings from the study is the increase in fiber abundance during S2 in various locations. Sites like Isola del Cavallo-Mezzocammino (Olbia farming area) with a peak of 27.3 ± 7.8 fibers per individual; Feraxi (Cagliari farming area) with 19.3 ± 5.9 fibers per individual, Tortolì (19.7 ± 3.7 fibers per individual), and Corru Mannu (Oristano farming area) with 19.33 ± 4.6 fibers per individual, showed a drastic rise in the number of items in the summer period compared to the spring (May) and winter (January) months. There may be many hypothetical causes of this summer peak. First, the increase in population due to tourist flows, including recreational boating. This aspect is particularly relevant for areas like Isola del Cavallo-Mezzocammino and Cugnana (Olbia farming area). From a purely physiological point of view, the increase in water temperature in summer also leads to an increase in the metabolic rate and filtration of mussels. A larger volume of filtered water means a higher chance of intercepting and trapping suspended particles in the water column. Additionally, local differences in water circulation patterns may have further influenced the differences documented in this study. Another seasonal factor to consider is the reduction in water exchange in lagoons or enclosed bays during the summer months, such as Corru Mannu (Oristano farming area), a phenomenon that can further promote the local accumulation of particles. On the other hand, at some sampling points like Cala Saccaia (Olbia farming area), the opposite trend was observed, with the highest levels recorded in S3 (January) with 11.67 fibers per individual. Since this is a predominantly industrial area, the winter increase could be linked to surface runoff caused by rain, which carries debris from the mainland to the sea, or to specific human activities not directly related to beach tourism. Comparing the average values of the individual sampling sites reveals differences that likely reflect the different land uses of the areas. We can therefore distinguish areas with high and low relative contamination, where average values recorded were respectively above and below 10 items per individual. The sampling points classified as highly contaminated, e.g., Isola del Cavallo-Mezzocammino, Golfo di Cugnana (Olbia farming area), Corru Mannu (Oristano farming area), Feraxi (Cagliari farming area) and Tortolì farming area, showed heterogeneous characteristics. Isola del Cavallo-Mezzocammino, Cugnana, Tortolì, or Feraxi are highly urbanized, especially during the summer season, due to the tourist appeal of the surrounding areas. On the contrary, in sampling sites like Corru Mannu and Terza Peschiera, the basin’s morphology and current circulation patterns may have locally favored particle accumulation. Among the sampling points with moderate or low contamination were classified Capo San Marco (average 5 ± 0.8 particles per individual) and Stagno di San Giovanni (7.14 particles per individual).

#### 3.3.3. Relations Between Biometry and Contamination

Our results showed a complex relationship between the size of the individual and fiber accumulation, which sometimes deviates from the positive correlation between the size of the individual and the volume of water filtered per unit of time. While some sampling points such as Torregrande (Oristano farming area), with 9.17 cm of total width and 11.33 particles per individual in S1, and Santa Gilla 2 (Cagliari farming area), with 5.27 cm of total width and 5.00 particles per individual in S2, seem to support this hypothesis, other sampling points such as Isola del Cavallo-Mezzocammino (Olbia farming area) showed a different trend. In S2, medium-sized individuals (7.47 cm total width) showed the highest absolute load with 27.33 particles per individual. This evidence suggests that the size of the individual is likely a predisposing factor; however, the environmental load, meaning the amount of plastic available in the water, could be the main factor. We can therefore assume that, in case of particularly high environmental contamination, this level could increase regardless of the biometric characteristics of the individuals.

### 3.4. Characterization of MFs by Fourier Transform Infrared Microscopy (μ-FTIR)

Polymeric characterization through ATR-µFTIR was carried out on a representative sub-sample (10%) of all the collected MFs, consisting of the larger ones (*n* = 100). The qualitative analysis showed that the two predominant polymers, which together make up over 85% of the MFs isolated from *Mytilus galloprovincialis* individuals, were natural and therefore not synthetic (Table 4). Previous extensive studies carried out in the Mediterranean area identified plastic polymers such as polypropylene (PP), polystyrene (PS), and polyvinyl chloride (PVC) as the most commonly found types of plastics in *Mytilus galloprovincialis* individuals [24]. The dominant polymer was cellulose, which accounted for 55.29% of the total (Figure 3), followed by cotton (30.59%). As for synthetic polymers, these made up only a minor fraction (~15% of the total), with polypropylene (PP) being the most abundant (5.88%), followed by polyethylene terephthalate (PET), polyethylene (PE) and polyester (1.18% each). The predominance of fibers at all the farming areas is consistent with what has been reported in the scientific literature for coastal surface waters of the Mediterranean basin [37,38], and more specifically in studies where *Mytilus galloprovincialis* was used as a target organism [29,39]. The average fiber length varied considerably, from high values of ~14.77 mm found in Feraxi (Cagliari farming area) and 12.10 mm found in the Gulf of Cugnana (Olbia farming area) to lower values of 4.09 mm highlighted at Santa Gilla 3 (Cagliari farming area). The high incidence of naturally derived materials like cellulose and cotton is consistent with results from studies on MP contamination in oceanic and Mediterranean surface waters [40]. We can therefore suppose that urban wastewater (e.g., discharge from home washing machines or dryers) could hypothetically be the main source of contamination.

### 3.5. Quantification of Trace Elements in MPs Extracted from Mytilus galloprovincialis

The concentrations of trace elements in MPs extracted from *Mytilus galloprovincialis* are summarized in Table 5. For each farming area, the mean values of the twenty-two heavy metals (Be, Tl, Sb, Ag, Hg, Th, U, Sn, Co, Cd, Li, Mo, Cr, Ba, V, Ni, Pb, Se, Mn, Cu, As, Zn) are reported. Most of the essential and non-essential trace elements showed to be below the method’s limit of quantification (LOQ) (0.010 mg·kg^−1^) in all the farming areas. The heavy metals that showed slight contamination in the MPs fraction were Sb (Oristano farming area), Cu (Cagliari farming area), Mn and Zn in all four farming areas, although the concentrations were very low and significantly lower than the metal contamination associated with the mussel itself. This evidence suggests that MPs represents a negligible source of trace elements compared to the main biological matrix.

## 4. Conclusions

This is the first study to evaluate the role of MPs as vectors of heavy metal pollution in the Sardinian coastal environment (Italy) and to contribute to the assessment of consumer exposure to MPs and heavy metals associated with the consumption of *Mytilus galloprovincialis*. The results of our investigation provide helpful data on the presence of essential and non-essential elements in Mediterranean mussels and highlighted that all samples were compliant with the European Union official limits set for Hg, Cd and Pb [35]. A widespread MF contamination was pointed out, albeit with marked spatial and temporal variability. Among the investigated farming areas, Capo San Marco (Oristano farming area) exhibited the lowest average abundance of fibers, whereas Isola del Cavallo-Mezzocammino (Olbia farming area) showed both the highest overall levels and peak fiber accumulation during S2. This peak is likely related to increased human activity, including tourism and recreational boating, as well as seasonal factors such as elevated water temperatures during summer, which boost mussel metabolism and filtration rates. The detection of fibers at all surveyed sites supports the use of *Mytilus galloprovincialis* as a reliable bioindicator of plastic particles and MPs pollution. Heavy metal concentrations associated with MPs ingested by *Mytilus galloprovincialis* were markedly lower than those measured directly in the edible tissues of the mussels. This indicates that MPs likely contribute minimally as vectors of metals to human consumers, especially when compared with the metals naturally bioaccumulated by the organisms. Consequently, the presence of MPs in Mediterranean mussels does not appear to represent a significant risk in terms of metal exposure. Nevertheless, it is important to highlight that further research, involving a larger number of samples and fully standardized methodologies, is needed to confirm these findings and to evaluate potential environmental or seasonal variations.

## Figures and Tables

**Figure 1:**
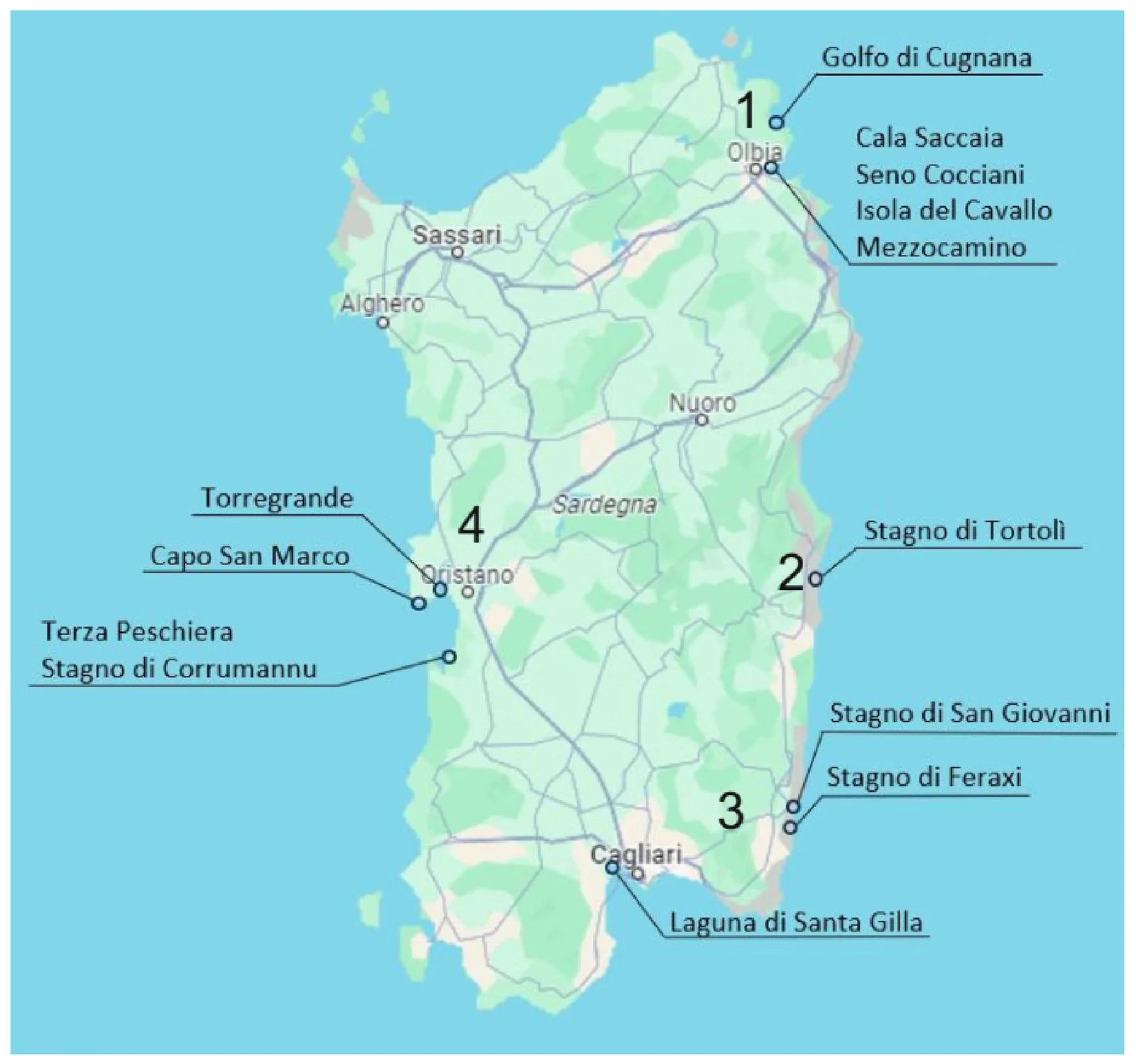
Area of study reporting the four farming areas (1–4) and the respective sampling stations.

**Figure 2:**
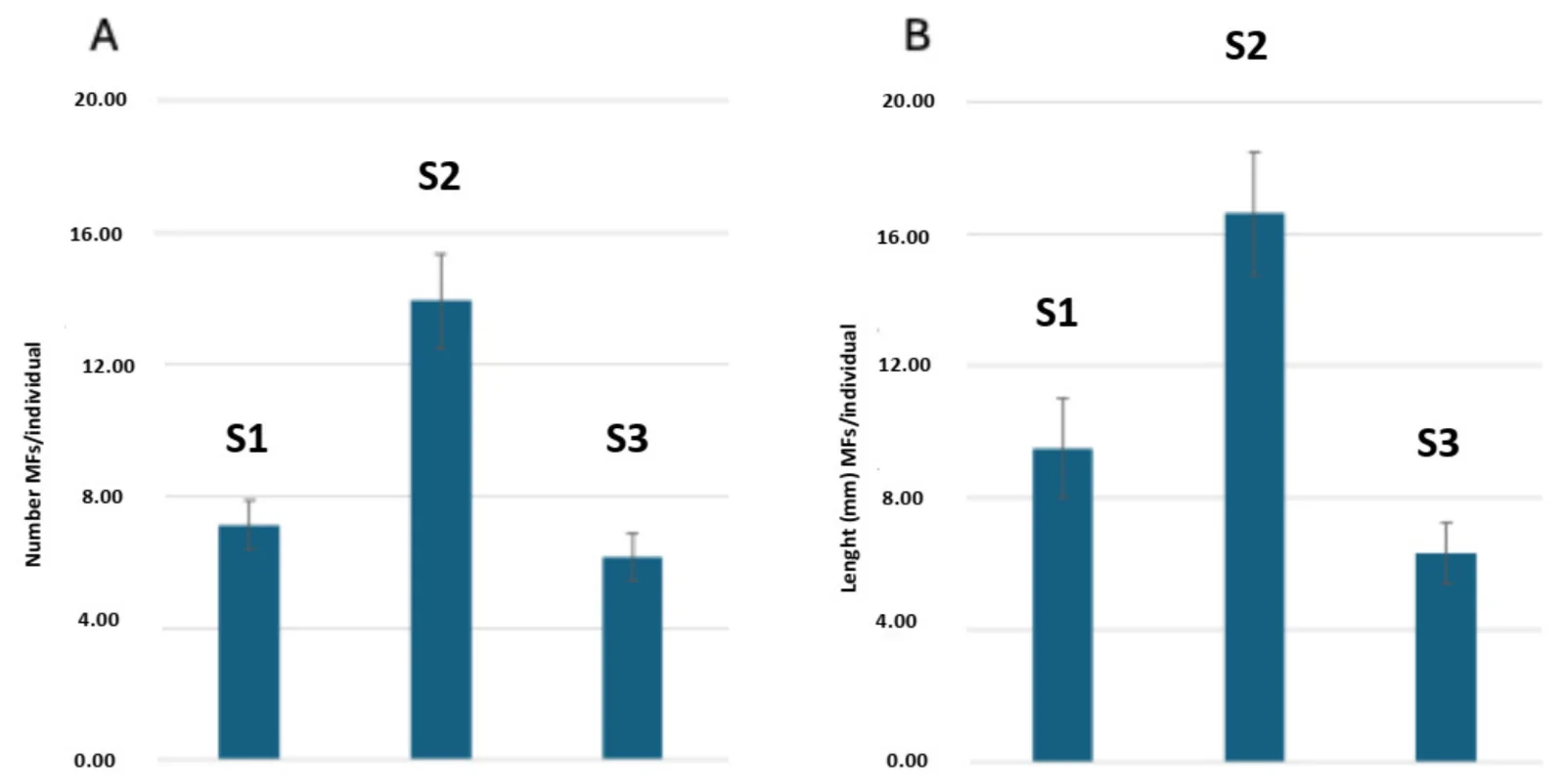
(**A**) Mean number of MFs per mussel. (**B**) Average length of the MFs (mm) over the three samplings: S1-S2-S3.

**Figure 3:**
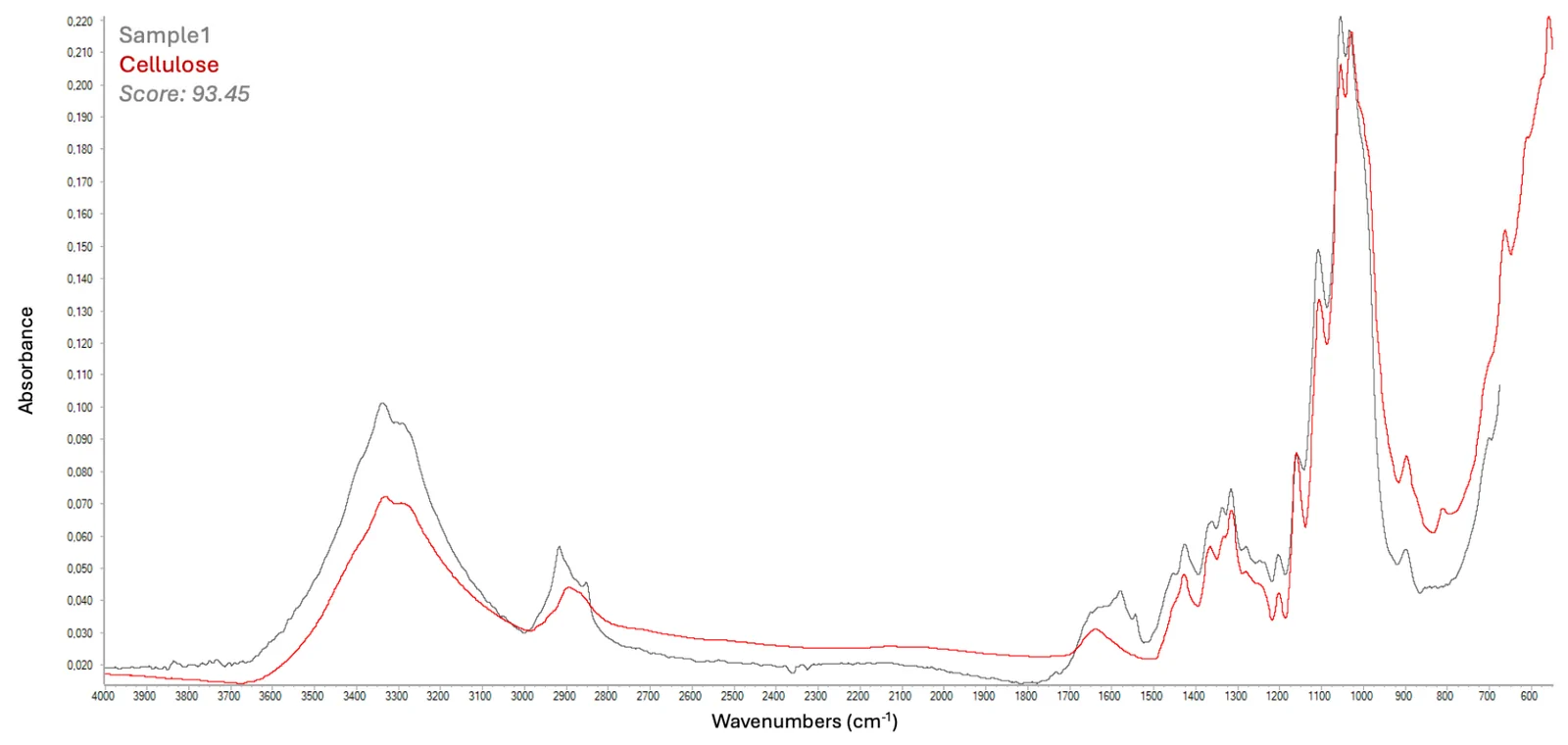
Cellulose fiber (μ-FTIR spectrum) detected in *Mytilus galloprovincialis* samples.

**Table 1:** Morphology (mean ± standard deviation) of *Mitylus galloprovincialis* samples analyzed in this study.

Farming Area	Sampling Point	Total Length (cm)	Total Width (cm)	Total Weight (g)	Soft Tissue Weight (g)
Olbia	Cala Saccaia	7.09 ± 0.30	3.63 ± 0.18	22.71 ± 2.46	4.12 ± 0.48
	Isola del Cavallo-Mezzocammino	7.56 ± 0.15	3.90 ± 0.10	25.01 ± 2.14	6.99 ± 0.89
	Seno Cocciani	7.17 ± 0.22	3.52 ± 0.10	23.67 ± 2.80	5.92 ± 1.16
	Golfo di Cugnana	6.72 ± 0.33	3.61 ± 0.14	14.16 ± 2.24	3.69 ± 0.81
Tortolì	Stagno di Tortolì	6.83 ± 0.17	3.39 ± 0.11	15.64 ± 1.74	4.64 ± 0.37
Cagliari	Laguna di Santa Gilla 2	5.70 ± 0.29	3.26 ± 0.15	10.84 ± 0.92	3.40 ± 0.38
	Laguna di Santa Gilla 3	6.57 ± 0.24	3.58 ± 0.23	21.00 ± 2.34	5.58 ± 1.29
	Stagno di San Giovanni	6.38 ± 0.19	3.56 ± 0.18	14.34 ± 1.05	4.44 ± 0.61
	Stagno di Feraxi	7.32 ± 0.16	3.83 ± 0.14	19.71 ± 1.83	5.44 ± 0.69
Oristano	Capo San Marco	6.96 ± 0.28	3.62 ± 0.16	19.77 ± 2.43	5.10 ± 0.68
	Stagno di Corru Mannu	7.10 ± 0.24	3.70 ± 0.16	17.82 ± 2.50	4.32 ± 0.74
	Torregrande	7.76 ± 0.41	3.92 ± 0.18	27.42 ± 4.20	5.63 ± 0.63
	Terza Peschiera	7.08 ± 0.26	3.67 ± 0.20	21.59 ± 2.04	5.52 ± 0.39

**Table 2:** Mean values of trace elements in *Mytilus galloprovincialis* from the four farming areas (mg·kg^−1^).

Farming Area	Be	Tl	Sb	Ag	Hg	Th	U	Sn	Co	Cd	Li	Mo	Cr	Ba	V	Ni	Pb	Se	Mn	Cu	As	Zn
Olbia	- *	-	-	-	-	0.020	0.016	0.070	0.066	0.080	0.150	0.186	0.141	0.123	0.142	0.640	0.130	0.311	1.420	2.048	1.810	28.623
Tortolì	-	-	-	-	-	-	-	0.022	0.076	0.067	0.046	0.122	0.075	0.086	0.167	0.162	0.136	0.359	0.975	0.960	1.947	18.875
Cagliari	-	-	-	-	-	0.012	0.011	0.050	0.082	0.079	0.076	0.092	0.145	0.981	0.144	0.182	0.330	0.403	1.374	2.140	1.517	24.531
Oristano	-	-	-	-	-	-	0.013	0.062	0.071	0.088	0.088	0.156	0.125	0.168	0.169	0.151	0.188	0.335	1.000	1.582	1.884	31.493

* <LOQ = below limit of quantification (0.010 mg·kg^−1^).

**Table 3:** Microfibers (MFs) abundance and frequency of occurrence (mean ± standard deviation) in *Mitylus galloprovincialis* samples analyzed in this study.

Farming Area	Sampling Point	Total Number of MFs	Mean Number of MFs per Mussel	Sum of the Lengths of the MFs (mm)	Average Length of the MFs (mm)
Olbia	Cala Saccaia	74	8.2 ± 1.2	63.9	7.1 ± 1
	Isola del Cavallo-Mezzocammino	111	12.33 ± 4.42	122.97	13.66 ± 5.64
	Seno Cocciani	83	9.22 ± 1.83	88.778	9.86 ± 2.81
	Golfo di Cugnana	90	10 ± 1.84	108.94	12.1 ± 2.6
Tortolì	Stagno di Tortolì	95	10.5 ± 2.6	91.8	10.2 ± 3.2
Cagliari	Laguna di Santa Gilla 2	78	8.67 ± 2.35	132.352	14.71 ± 5.22
	Laguna di Santa Gilla 3	27	4.50 ± 1.67	24.52	4.1 ± 1.5
	Stagno di San Giovanni	50	7.14 ± 1.26	73.268	10.47 ± 3.03
	Stagno di Feraxi	91	10.1 ± 2.9	132.9	14.8 ± 4.1
Oristano	Capo San Marco	46	5 ± 0.80	44.7	4.9 ± 0.5
	Stagno di Corru Mannu	93	10.3 ± 2.8	79.35	8.8 ± 2.3
	Torregrande	80	8.9 ± 2.2	99.5	11.1 ± 3.1
	Terza Peschiera	89	9.89 ± 1.74	131.361	14.60 ± 3.18

**Table 4:** Composition of the 100 MFs isolated from Mytilus galloprovincialis specimens identified by Fourier Transform Infrared Microscopy (μ-FTIR).

Number	Shape	Composition
56	MF	cellulose
31	MF	cotton
6	MF	polypropylene (PP)
2	MF	wool
2	MF	wood
1	MF	polyethylene (PE)
1	MF	polyester
1	MF	poly (ethylene-terephthalate) (PET)

**Table 5:** Mean values of trace elements in MPs extracted from *Mytilus galloprovincialis* sampled in the four farming areas (mg·kg^−1^).

Farming Area	Be	Tl	Sb	Ag	Hg	Th	U	Sn	Co	Cd	Li	Mo	Cr	Ba	V	Ni	Pb	Se	Mn	Cu	As	Zn
Olbia	- *	-	-	-	-	-	-	-	-	-	-	-	-	-	-	-	-	-	0.025	-	-	0.096
Tortolì	-	-	-	-	-	-	-	-	-	-	-	-	-	-	-	-	-	-	0.065	-	-	0.088
Cagliari	-	-	-	-	-	-	-	-	-	-	-	-	-	-	-	-	-	-	0.087	0.012	-	0.101
Oristano	-	-	0.010	-	-	-	-	-	-	-	-	-	-	-	-	-	-	-	0.028	-	-	0.114

* <LOQ = below limit of quantification (0.010 mg·kg^−1^).

## Data Availability

The original contributions presented in this study are included in the article. Further inquiries can be directed to the corresponding author.
